# A novel pyruvate kinase and its application in lactic acid production under oxygen deprivation in *Corynebacterium glutamicum*

**DOI:** 10.1186/s12896-016-0313-6

**Published:** 2016-11-16

**Authors:** Xin Chai, Xiuling Shang, Yu Zhang, Shuwen Liu, Yong Liang, Yun Zhang, Tingyi Wen

**Affiliations:** 1 CAS Key Laboratory of Pathogenic Microbiology and Immunology, Institute of Microbiology, Chinese Academy of Sciences, Beijing, 100101 People’s Republic of China; 2 University of Chinese Academy of Sciences, Beijing, 100049 People’s Republic of China; 3 Savaid Medical School, University of Chinese Academy of Sciences, Beijing, 100049 People’s Republic of China

**Keywords:** *Corynebacterium glutamicum*, Pyruvate kinase, Oxygen deprivation, Lactate dehydrogenase, Fructose 1,6-bisphosphate

## Abstract

**Background:**

Pyruvate kinase (Pyk) catalyzes the generation of pyruvate and ATP in glycolysis and functions as a key switch in the regulation of carbon flux distribution. Both the substrates and products of Pyk are involved in the tricarboxylic acid cycle, anaplerosis and energy anabolism, which places Pyk at a primary metabolic intersection. Pyks are highly conserved in most bacteria and lower eukaryotes. *Corynebacterium glutamicum* is an industrial workhorse for the production of various amino acids and organic acids. Although *C. glutamicum* was assumed to possess only one Pyk (*pyk1*, NCgl2008), NCgl2809 was annotated as a pyruvate kinase with an unknown role.

**Results:**

Here, we identified that NCgl2809 was a novel pyruvate kinase (*pyk2*) in *C. glutamicum*. Complementation of the WTΔ*pyk1*Δ*pyk2* strain with the *pyk2* gene restored its growth on d-ribose, which demonstrated that Pyk2 could substitute for Pyk1 *in vivo*. Pyk2 was co-dependent on Mn^2+^ and K^+^ and had a higher affinity for ADP than phosphoenolpyruvate (PEP). The catalytic activity of Pyk2 was allosterically regulated by fructose 1,6-bisphosphate (FBP) activation and ATP inhibition. Furthermore, *pyk2* and *ldhA*, which encodes l-lactate dehydrogenase, were co*-*transcribed as a bicistronic mRNA under aerobic conditions and *pyk2* deficiency had a slight effect on the intracellular activity of Pyk. However, the mRNA level of *pyk2* in the wild-type strain under oxygen deprivation was 14.24-fold higher than that under aerobic conditions. Under oxygen deprivation, *pyk1* or *pyk2* deficiency decreased the generation of lactic acid, and the overexpression of either *pyk1* or *pyk2* increased the production of lactic acid as the activity of Pyk increased. Fed-batch fermentation of the *pyk2*-overexpressing WTΔ*pyk1* strain produced 60.27 ± 1.40 g/L of lactic acid, which was a 47% increase compared to the parent strain under oxygen deprivation.

**Conclusions:**

Pyk*2* functioned as a pyruvate kinase and contributed to the increased level of Pyk activity under oxygen deprivation.

**Electronic supplementary material:**

The online version of this article (doi:10.1186/s12896-016-0313-6) contains supplementary material, which is available to authorized users.

## Background

Pyruvate kinase catalyzes the transphosphorylation from PEP to ADP to generate pyruvate and ATP in glycolysis [1]. Pyks are highly conserved in both eukaryotes and prokaryotes [2, 3]. In mammals, four types of Pyk isoenzymes (M1, M2, L and R) with different kinetic properties are distributed in various tissues to satisfy the diverse metabolic requirements. Most bacteria and lower eukaryotes have only one form of Pyk. However, there are two identified Pyk isoenzymes in *Escherichia coli, Salmonella typhimurium* and *Saccharomyces cerevisiae*. The activities of Pyks are dependent on a bivalent and/or a univalent cation (Mn^2+^/Mg^2+^ and/or K^+^) [1]. In the majority of organisms, Pyk is a homotetramer that exhibits inactive/active reversible allosteric mechanisms [4]. The functional forms of Pyks are divided into the following two broad types based on their allosteric effectors: type I is allosterically activated by FBP, whereas type II is allosterically activated by AMP and monophosphorylated sugars [1]. The allosteric behavior of Pyk allows it to be an important switch point for the regulation of carbon flux distribution.

The two most studied allosteric Pyk isoenzymes are those of *E. coli* and *S. cerevisiae*. In *E. coli*, PykF exhibited higher activity than PykA *in vitro*, and PykF and PykA played different roles in pyruvate synthesis and cell growth [5–7]. In *S. cerevisiae,* FBP-activated Pyk1p was the major enzyme that catalyzed the conversion of PEP to pyruvate, whereas Pyk2p with relatively low enzymatic activity was insensitive to FBP and subject to glucose repression, which suggested that Pyk2p played a major role under conditions of low glycolytic flux [8]. To date, little is known about other Pyk isoenzymes and their physiological function in other bacteria.

*Corynebacterium glutamicum* is widely used for the industrial production of various amino acids and vitamins during aerobic cultivation due to the robustness of its genetic modifications and fermentation [9]. Under oxygen deprivation, *C. glutamicum* can primarily convert glucose to pyruvate, which is an intermediate for the production of organic acids and amino acids despite the cessation of growth [10–13]. Two genes [*NCgl2008* (*pyk1*) and *NCgl2809* (*pyk2*)] were annotated as pyruvate kinases in the genome of *C. glutamicum* ATCC 13032 [9]. The deletion of *pyk1* resulted in marginal Pyk activity that was below the detection limit, which supported the assertion that *C. glutamicum* possessed only one pyruvate kinase [14, 15]. Pyk1 exhibited high catalytic activity using Mn^2+^ or Co^2+^ as a cation and subjected to AMP activation and ATP inhibition [16]. The expression of *pyk1* was repressed by the global transcriptional regulator SugR, which binds to the promoters of many target genes involved in glycolysis (i.e., *gapA*, *pfkA*, *fba*, *eno, pyk1* and *ldhA*) to regulate sugar metabolism [17, 18]. The deletion of *pyk1* led to decrease biomass and increase glutamic acid production [14]. Therefore, Pyk1 functioned as an effective metabolic engineering node for the production of various metabolites [19]. The genetic organization of the homologous *pyk2*, which is adjacent to *ldhA*, exists in many *Corynebacterium* species. However, the function and biological significance of Pyk2 has not been understood.

In this study, we identified NCgl2809 (Pyk2) as a functional pyruvate kinase in *C. glutamicum*. Pyk2 exhibited different enzymatic properties compared to Pyk1 and could substitute for Pyk1 *in vivo*. Under oxygen deprivation, *pyk1* or *pyk2* deficiency decreased the generation of lactic acid, and the overexpression of either *pyk1* or *pyk2* increased the production of lactic acid.

## Results

### Pyk2 is a pyruvate kinase in *C. glutamicum*

*NCgl2809* was annotated as a pyruvate kinase in the genome of *C. glutamicum* ATCC 13032 (WT). This gene is adjacent to *ldhA* and distant from *pyk1* (Additional file 1: Figure S1a). A protein homology search and alignment indicated that Pyk2 had moderate sequence identity to the functionally identified Pyk1 of *C. glutamicum* (32%), PykF of *E. coli* (30%) and PykA of *E. coli* (27%), but showed high sequence identity to the annotated homologs from other species of *Corynebacterium.* Using the PykF crystal structure from *E. coli* (PDB code 1PKY) as a template, the secondary structure of Pyk2, which ranged from 128 to 213 residues and 346 to 606 residues, consisted of 12 α-helices and 16 β-strands structures. This structure was similar to the catalytic domain A of PykF (Additional file 1: Figure S1b and c), in which the GDLGVE motif served as the PEP binding site and the conserved sequences assisted the FBP allosteric regulation. A long extra N-terminal sequence of 120 amino acids made Pyk2 (67.6 kDa) larger than other typical bacterial Pyks (51 kDa).

**Fig. 1:**
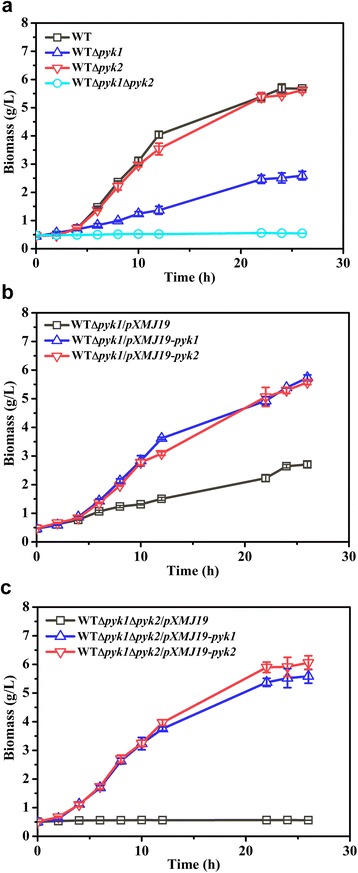
Growth of *C. glutamicum* strains on CGXII medium with 100 mM d-ribose under aerobic conditions. *C. glutamicum* strains were aerobically pre-cultivated in 50 mL of CGIII medium. After washing the cells in CGXII minimal medium, the cultures were inoculated to obtain optical densities at 600 nm of approximately 1.5 (dry cell weight of 0.45 g/L). When necessary, 0.5 mM IPTG was supplemented after inoculation. **a** WT, WT∆*pyk1*, WT∆*pyk*2 and WT∆*pyk1*∆*pyk2*. **b** WT∆*pyk1*/pXMJ19, WT∆*pyk1*/pXMJ19-*pyk1* and WT∆*pyk1*/pXMJ19-*pyk2*. **c** WT∆*pyk1*∆*pyk2*/pXMJ19, WT∆*pyk1*∆*pyk2*/pXMJ19-*pyk1* and WT∆*pyk1*∆*pyk2*/pXMJ19-*pyk2*. The data were derived from experiments performed at least in three times, and the *error bars* represent the standard deviations

To demonstrate the function of Pyk2 *in vivo*, the *pyk2* and/or *pyk1* genes were deleted in the chromosome. The WTΔ*pyk1*Δ*pyk2* strain grown on glucose under aerobic conditions exhibited only a slightly lower growth rate than the WT strain (*P* < 0.05), which indicated that the conversion of PEP to pyruvate during glucose transport by the phosphotransferase transport system (PTS) could supply pyruvate for cell growth (Additional file 1: Table S1). To eliminate the effect of the pyruvate generated from the glucose transport on growth, d-ribose, which was imported by the ribose-specific ATP-binding cassette transport system, was employed as the sole carbon source to evaluate the role of Pyk isoenzymes under aerobic conditions [20]. As shown in Fig. 1a, the WTΔ*pyk2* strain showed similar growth compared to the WT strain. However, the WTΔ*pyk1* strain showed a 52% decrease in the final biomass and the WTΔ*pyk1*Δ*pyk2* strain failed to grow when d-ribose was the sole carbon source, which suggested that the *pyk2* gene maintained cell growth on d-ribose to some extent. Moreover, complementation of the WTΔ*pyk1* strain with the *pyk2* gene restored its growth in the same manner as the *pyk1* gene (Fig. 1b), which indicated that Pyk2 could substitute for Pyk1 in *C. glutamicum*. Furthermore, complementation of the WTΔ*pyk1*Δ*pyk2* strain with *pyk1* and *pyk2* completely restored its growth (Fig. 1c). In addition, the overexpression of *pyk2* in the WTΔ*pyk1*Δ*pyk2* strain made the specific activities of Pyk (448.81 ± 19.25 mU/mg) comparable to that of overexpression of *pyk1* (458.76 ± 7.99 mU/mg) in the WTΔ*pyk1*Δ*pyk2* strain. Altogether, our results strongly supported that Pyk2 functioned as a pyruvate kinase *in vivo*.

### Enzymatic properties of Pyk2

**Fig. 2:**
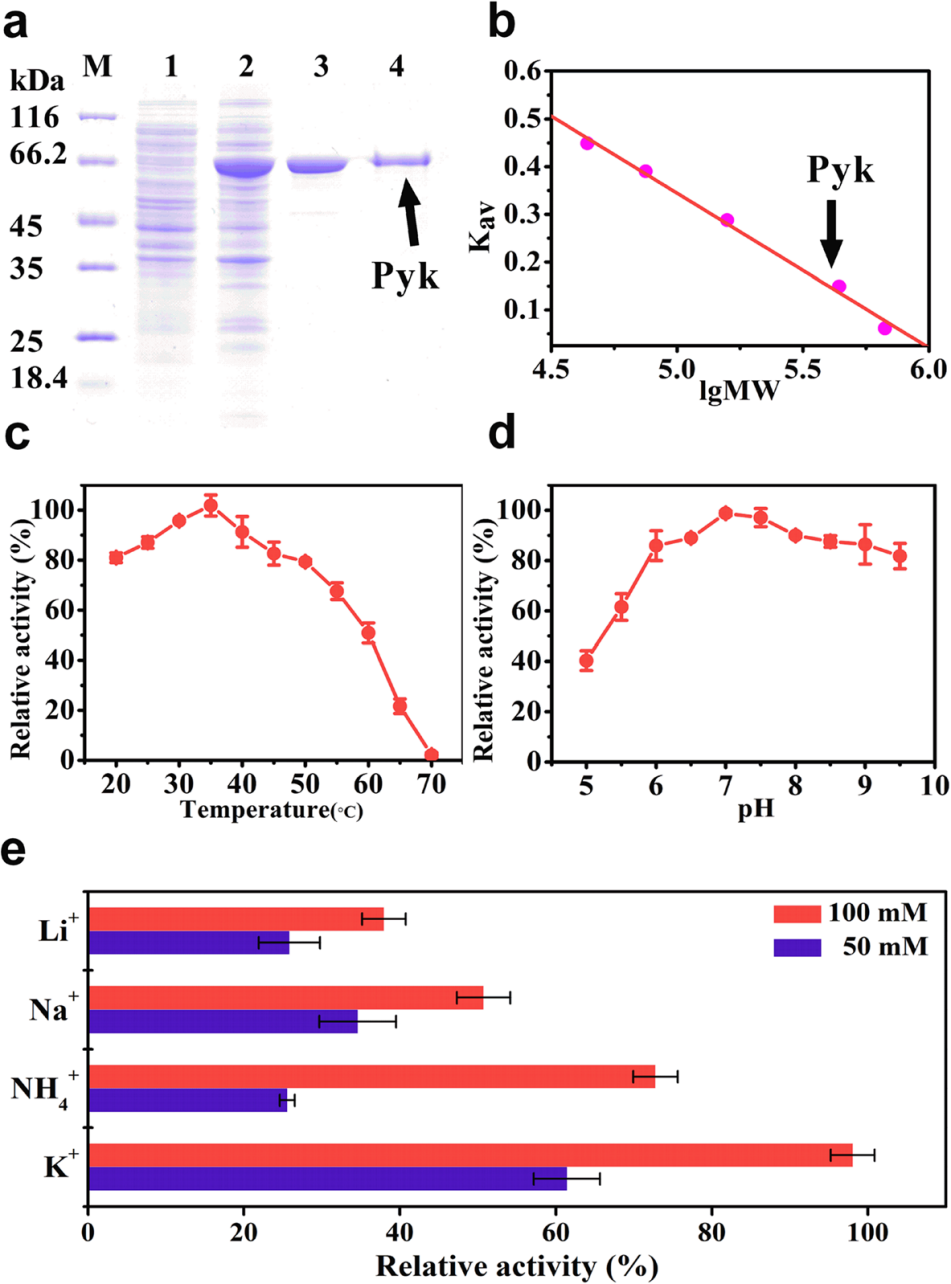
Enzymatic properties of Pyk2 from *C. glutamicum*. **a** SDS-PAGE of purified Pyk2. Lane 1, supernatant of crude extract from *E. coli* BL21(DE3)/pET-28a; Lane 2, supernatant of crude extract from *E. coli* BL21(DE3)/pET-28a-*pyk2*; Lane 3, Pyk2 purified by His-tagged affinity chromatography; and Lane 4, Pyk2 purified by ion exchange chromatography. **b** Molecular weight determination by gel filtration chromatography. The molecular weight of a protein could be determined from the calibration curve (plot of K_av_ versus the logarithm of molecular weight). The K_av_ value was calculated from the measured elution volume using the following equation: K_av_ = (V_e_ − V_o_)/(V_c_ − V_o_), where V_e_ is the elution volume, V_o_ is the column void volume, and V_c_ is the geometric column volume. The logarithm of the molecular weight was represented as lgMW. The standard proteins were shown as follows (●): 1, ovalbumin (44 kDa); 2, conalbumin (75 kDa); 3, aldolase (158 kDa); 4, ferritin (440 kDa); and 5, thyroglobulin (669 kDa). The native molecular weight of Pyk2 was determined to be a homohexamer of 430,881 (*arrow*). **c** The enzyme activity of Pyk2 at different temperatures. The maximum value of 2.19 ± 0.09 U/mg was set as relative 100%. **d** Enzyme activities of Pyk2 at various pHs (The maximum value of 2.43 ± 0.02 U/mg was set as relative 100%). **e** Enzyme activities of Pyk2 in the presence of various monovalent ions (The maximum value of 1.77 ± 0.05 U/mg was set as relative 100%). The enzyme was incubated in 100 mM Tris-HCl buffer (pH 7.4) and 5 mM Mn^2+^ with 50 mM or 100 mM monovalent ions. The data were derived from at least three replicate experiments, and the *error bars* represent the standard deviations

To detect the activity of Pyk2 *in vitro*, the *pyk2* gene of *C. glutamicum* was expressed in *E. coli* BL21 (DE3) using the pET-28a expression system. N-terminal His-tagged Pyk2 exhibited similar activity to Pyk2 with the His-tag removed by thrombin cleavage. After further purification using ion exchange chromatography, the recombinant Pyk2 showed an approximately 12.81-fold purification with a yield of 37% and a specific activity of 1.64 U/mg. The molecular weight of the recombinant Pyk2 subunit was 67,572 Da (Fig. 2a). Gel filtration chromatography yielded a 430,881 Da fraction that corresponded to the predicted size of a homohexamer, which was different from the homotetramer observed for other Pyks (Fig. 2b).

Pyk2 was active at temperatures between 20 and 50 °C, with a maximal activity at 35 °C (Fig. 2c). The enzyme maintained greater than 80% of its relative activity at a pH range between 6 and 9, with a maximal activity at pH 7.0 (Fig. 2d). Pyk2 exhibited an absolute dependence on Mn^2+^ together with a monovalent cation. This finding was quite different from Pyk1 in *C. glutamicum*, which only used Mn^2+^ or Co^2+^ as cations [16]. Among all the tested monovalent cations, the maximum activity of Pyk2 was detected in the presence of 5 mM Mn^2+^ and 100 mM K^+^. When NH_4_^+^, Na^+^, or Li^+^ was substituted for K^+^, the relative activities of Pyk2 were maintained at 73, 51, and 38%, respectively (Fig. 2e).

### Kinetic parameters of Pyk2

**Fig. 3:**
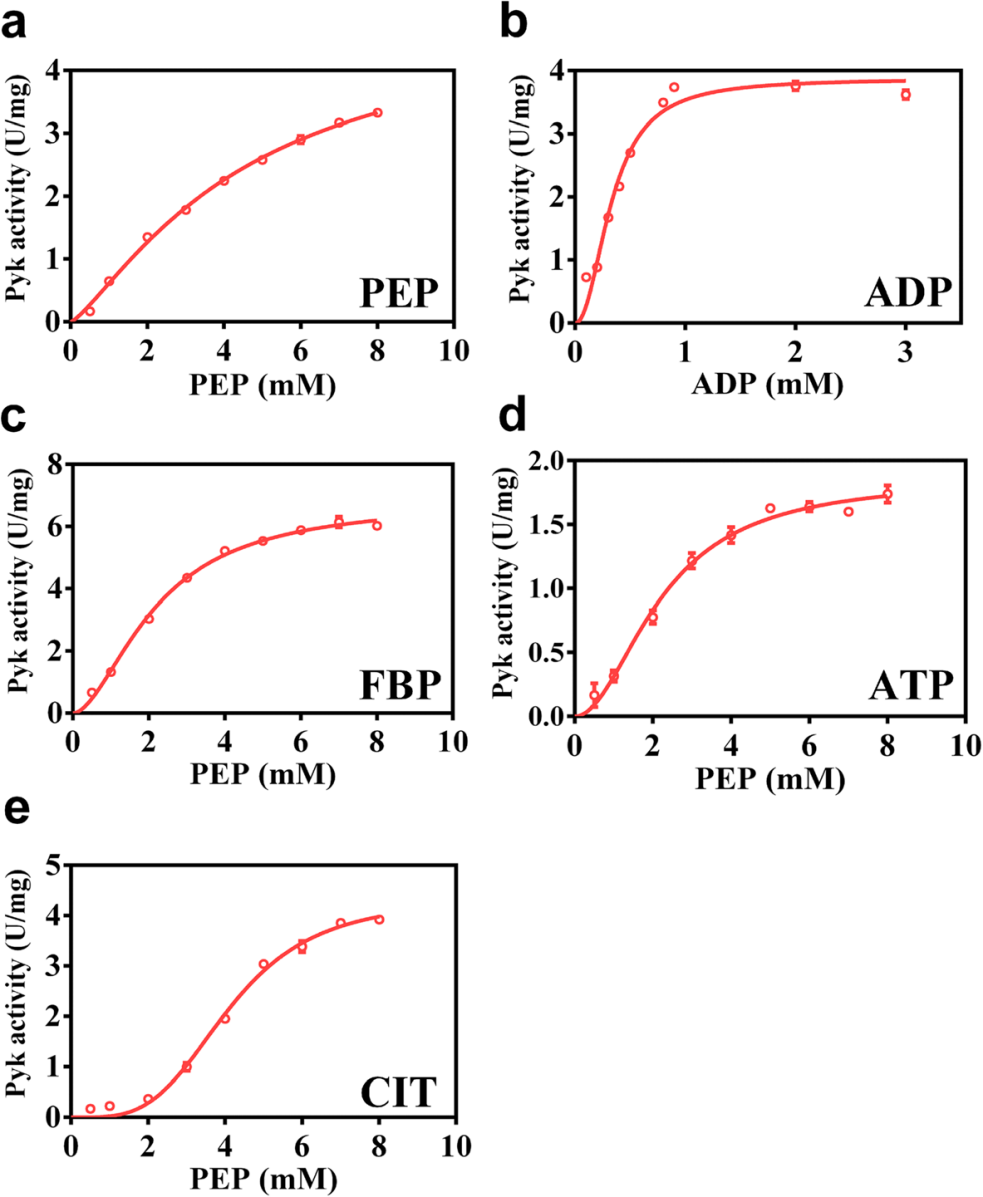
Kinetic parameters of Pyk2. The kinetic parameters of Pyk2 with respect to the substrates are shown. **a** PEP. **b** ADP. The kinetic parameters of Pyk2 with respect to PEP at a concentration of 5 mM different effectors are shown. **c** FBP. **d** ATP. **e** CIT. The data points were fitted to the allosteric-sigmoidal equation using Prism software (GraphPad, USA). The *S*_0.5_ (the substrate concentration giving one-half of the *V*_max_) and *k*_*cat*_ values were calculated. The experiments were performed at least in three times, and the *error bars* represent the standard error of the regression

The kinetics of Pyk2 were determined with respect to PEP and ADP as described in the Methods section (Fig. 3 and Additional file 1: Table S2). As shown in Table S2, the *S*_0.5_ value of Pyk2 for PEP was 4.58 ± 0.74 mM, and the *k*_*cat*_ value was 5.58 ± 0.53 s^−1^, which was lower than that of Pyk1 [16]. The *k*_*cat*_ value of Pyk2 for ADP was 4.36 ± 0.22 s^−1^, and the *S*_0.5_ value was 0.33 ± 0.03 mM (Fig. 3b); this latter value was 4.71-fold higher than that of *C. glutamicum* Pyk1 (0.07 mM), which indicated that Pyk2 had a relatively lower affinity for ADP compared to Pyk1.

The kinetic properties of Pyk2 in response to different effectors were determined to understand its allosteric regulation. In the presence of 5 mM FBP (Fig. 3c), the *S*_0.5_ of Pyk2 for PEP was dramatically decreased to 2.17 ± 0.12 mM and the *k*_*cat*_ value was increased to 7.67 ± 0.26 s^−1^, which demonstrated that Pyk2 was dependent on FBP activation. However, glucose-6-phosphate (G6P) had no significant effect on the *S*_0.5_ and *k*_*cat*_ of Pyk2 (Additional file 1: Figure S2a). In addition, there was no significant effect of AMP on the *S*_0.5_ and *k*_*cat*_ of Pyk2 (Additional file 1: Figure S2b). The kinetic properties of Pyk2 were similar to *E. coli* PykF and different from *C. glutamicum* Pyk1, which was activated by AMP [1]. Moreover, ATP had an inhibitory effect on Pyk2 because the *k*_*cat*_ for PEP decreased to 2.09 ± 0.10 s^−1^ (Fig. 3d). In addition, in the presence of citrate (CIT), the sigmoidal nature of the activity curve increased to a Hill coefficient of 3.66 that was indicative of increased cooperativity (Fig. 3e). Altogether, the FBP-activated Pyk2 was different from the AMP-activated *C. glutamicum* Pyk1.

### The expression of *pyk2* is induced under oxygen deprivation

**Fig. 4:**
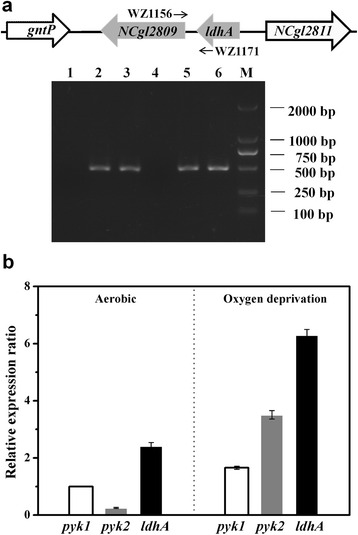
Expression of the *ldhA* and *pyk2* genes in *C. glutamicum*. **a** Identification of the co-transcription of *ldhA* and *pyk2* in the *ldhA-pyk2* cluster using RT-PCR. The *C. glutamicum* WT strain was cultured in minimal medium with glucose under aerobic conditions. The templates used for the PCR were as follows: lanes 1 and 4, total RNA reverse transcribed without reverse transcriptase; lanes 2 and 5, genomic DNA; and lanes 3 and 6, cDNA. The fragments in lanes 1, 2 and 3 were amplified using the primers WZ1181/WZ868 for *rpoB.* In addition, the primers WZ1171 and WZ1156 were used for the *ldhA-pyk2* region in lanes 4, 5 and 6. **b** The relative transcription levels of the *pyk1*, *pyk2* and *ldhA* genes were analyzed by qRT-PCR. Total RNA was isolated from WT cells harvested at the exponential phase under aerobic conditions and at 3 h cultivation under oxygen-deprived conditions. The expression levels of *pyk1*, *pyk2* and *ldhA* under different conditions were compared against the expression of *pyk1* under aerobic conditions (=1). The mean values from at least three independent cultures are shown with the standard deviations

Considering that the transcriptional direction of *pyk2* and the neighboring *ldhA* genes in the chromosome are similar, RT-PCR was performed using two primers designed against the overlapping adjacent region of the two genes to determine whether *pyk2* and *ldhA* were co-transcribed. Under aerobic conditions, a single 491 bp PCR product was obtained, which indicated that *pyk2* and *ldhA* were co-transcribed as a bicistronic mRNA (Fig. 4a). The expression levels of *pyk1*, *pyk2* and *ldhA* in the WT strain were then further investigated using qRT-PCR under aerobic and oxygen-deprived conditions. The mRNA level of *pyk2* under oxygen deprivation was 14.24-fold higher than that under aerobic conditions (Fig. 4b), which indicated that *pyk2* was induced at the transcription level under oxygen deprivation. The mRNA level of *ldhA* under oxygen deprivation was 2.61-fold higher than under aerobic conditions, which was consistent with the observed increase of *ldhA* expression in the WT strain under low oxygen tension [21]. In contrast, the mRNA level of *pyk1* under oxygen deprivation exhibited a 1.65-fold increase compared to the mRNA level obtained under aerobic conditions.

The Pyk activities in the WT, WTΔ*pyk1*, WTΔ*pyk2* and WTΔ*pyk1*Δ*pyk2* strains were investigated under aerobic and oxygen-deprived conditions. The Pyk activity in the WT strain under oxygen-deprived conditions increased by 12% compared to the activity under aerobic conditions, which was mainly attributed to the increased expression of *pyk2* (Table 1). The deficiency of *pyk1* resulted in a 97% decrease in Pyk activity; however, the Pyk activity in the WTΔ*pyk2* strain was comparable to that in the WT strain under aerobic conditions. In addition, there was no significant difference in the specific glucose consumption rate and the specific growth rate of the WTΔ*pyk2* strain compared with the WT strain (Additional file 1: Table S1), which indicated that the *pyk2* gene might maintain a relatively low expression level under aerobic conditions. In contrast, *pyk2* deficiency led to a 15% decrease in Pyk activity under oxygen-deprived conditions, and Pyk1 could only maintain 85% of Pyk activity in the WTΔ*pyk2* strain compared to the WT strain. Therefore, these results illustrated that the expression of *pyk2* was induced and contributed to increased whole cell Pyk activity under oxygen deprivation.

**Table 1:** The production of organic acids by *C. glutamicum* strains under oxygen deprivation^a^

Strains	Glucose (mM/h)	Lactic acid (mM)	Succinic acid (mM)	Acetic acid (mM)	Lactic acid Yield (%)^b^	Pyk activity (mU/mg)^c^
WT	28.46 ± 0.56	126.18 ± 1.70	22.59 ± 1.65	5.17 ± 0.05	73.90 ± 0.99	471.06 ± 6.61 (418.56 ± 6.54)*
WTΔ*pyk1*	27.72 ± 0.28	109.90 ± 4.00*	44.49 ± 0.88*	5.89 ± 0.91	66.09 ± 2.41*	69.98 ± 5.00* (11.34 ± 0.42)*
WTΔ*pyk2*	29.75 ± 0.11	117.30 ± 2.31*	38.02 ± 0.82*	5.35 ± 0.76	65.71 ± 1.29*	402.29 ± 8.98* (406.87 ± 14.69)
WTΔ*pyk1*Δ*pyk2*	28.83 ± 0.38	98.80 ± 1.11*	47.83 ± 0.86*	6.06 ± 0.64	57.12 ± 0.64*	ND (ND)
WTΔ*pyk1*Δ*pyk2*/pXMJ19	29.51 ± 0.43	99.17 ± 2.31*	49.45 ± 1.69*	4.40 ± 1.80	56.02 ± 1.30*	ND
WTΔ*pyk1*Δ*pyk2*/pXMJ19-*pyk1*	29.81 ± 0.85	127.29 ± 3.57	28.42 ± 3.13*	6.30 ± 2.19	71.16 ± 1.99	472.94 ± 21.29
WTΔ*pyk1*Δ*pyk2*/pXMJ19-*pyk2*	29.69 ± 1.20	128.77 ± 2.22	29.11 ± 5.13*	6.23 ± 2.07	72.28 ± 1.25	442.72 ± 10.64
WT/pXMJ19	29.63 ± 0.56	125.44 ± 4.00	27.59 ± 0.76*	6.72 ± 1.16	70.56 ± 2.25	486.38 ± 31.68
WT/pXMJ19-*pyk1*	32.78 ± 0.74*	150.98 ± 2.94*	13.37 ± 0.14*	12.78 ± 1.29*	76.77 ± 1.49*	926.55 ± 63.96*
WT/pXMJ19-*pyk2*	33.58 ± 0.28*	146.17 ± 3.20*	19.20 ± 1.27*	15.38 ± 0.89*	72.54 ± 1.59	911.45 ± 31.86*

^a^The cells were suspended to a final dry cell weight of 10 g/L in 100 mL of CGXII medium with 100 mM bicarbonate. The values were analyzed after 3 h under oxygen deprivation. The data were derived from experiments performed at least in three times, and the *error bars* represent the standard deviations

^b^The yields were calculated as a percentage of the theoretical yield (100% represented 2 mol of lactic acid per 1 mol of glucose consumed)

^c^Pyk activities were measured under oxygen deprivation as described above. And the values in parentheses show the Pyk activities at the exponential growth phase under aerobic conditions. ND, not detected

*,significant differences between WT and mutants under oxygen deprivation analyzed by *t*-test (*P* < 0.05) were marked. And the differences of Pyk activities of strains between aerobic and oxygen-deprived conditions are also shown (*P* < 0.05)

### The effect of *pyk1* or *pyk2* expression on the production of lactic acid under oxygen deprivation

Pyk2 had a slight effect on Pyk activity under aerobic conditions, and therefore, the role of Pyk2 was further investigated in the WT, WTΔ*pyk1*, WTΔ*pyk2* and WTΔ*pyk1*Δ*pyk2* strains under oxygen-deprived conditions. There was no obvious difference in the glucose consumption rate; however, a significant change was observed in the production of organic acids among these strains (Table 1). The yield of lactic acid from glucose in the *pyk1*- or *pyk2*-deficient strains was similar, but there was a 13 and 7% decrease in the production of lactic acid in the WTΔ*pyk1* and WTΔ*pyk2* strains, respectively, compared to the WT strain. The production of lactic acid in the double mutant decreased by 22%, and the yield further decreased by 23%. In contrast, the variation tendency of production of succinic acid was opposite to that of lactic acid. Compared to the WT strain, the production of succinic acid was 1.68 and 1.97-fold higher in the WTΔ*pyk2* and WTΔ*pyk1* strains, respectively. In addition, the titers of acetic acid were lower in the three mutants. These results indicated that *pyk1* or *pyk2* deficiency decreased the generation of lactic acid and increased the formation of succinic acid.

*Pyk1* or *pyk2* was overexpressed in the WTΔ*pyk1*Δ*pyk2* and WT strains to determine their effects on the production of organic acids. Complementation of the WTΔ*pyk1*Δ*pyk2* strain with *pyk1* and *pyk2* resulted in a lactic acid production that was comparable to that of the WT strain (Table 1). Pyk activity was restored to a similar level as the WT strain. Furthermore, the titers of acetic acid in the three mutants were not significantly different from those of the WT strain. However, the overexpression of the *pyk1* or *pyk2* gene produced a 20 and 17% increase in the production of lactic acid in the WT strain, with 1.90 and 1.87-fold higher Pyk activities, respectively. In addition, the production of succinic acid was significantly decreased, and the production of acetic acid was increased in the mutants. Therefore, the overexpression of either *pyk1* or *pyk2* increased the production of lactic acid under oxygen deprivation.

### Fed-batch lactic acid production of the *pyk2*-overexpressing strain

**Fig. 5:**
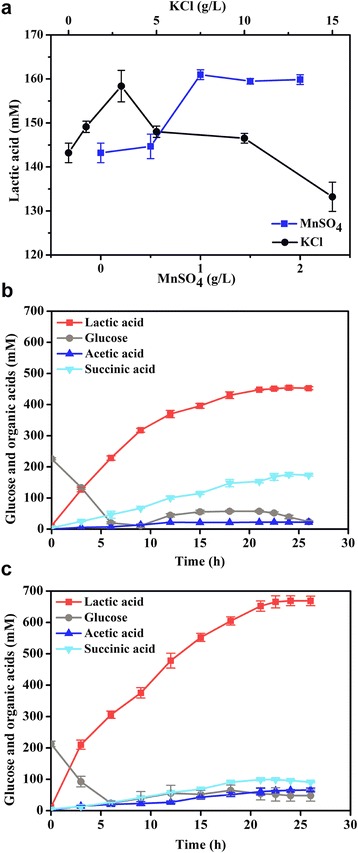
Lactic acid production by *C. glutamicum* strains under oxygen deprivation. **a** The effects of MnSO_4_ and KCl on lactic acid production by the WT∆*pyk1*/pXMJ19-*pyk2* strain. The cells were suspended to a final dry cell weight of 10 g/L in 100 mL of CGXII medium with 100 mM bicarbonate. **b** Fed-batch production by the WT∆*pyk1* strain under oxygen deprivation. **c** Fed-batch production by the WT∆*pyk1*/pXMJ19-*pyk2* strain under oxygen deprivation. For the fed-batch fermentation experiments, the cells were suspended to a final dry cell weight of 20 g/L in 3 L of CGXII medium with 100 mM bicarbonate. The data were derived from experiments performed at least in three times, and the *error bars* represent the standard deviations

Because both Mn^2+^ and K^+^ are necessary for Pyk2 activity, the effects of the concentrations of MnSO_4_ and KCl in the medium on the production of lactic acid were investigated in the WTΔ*pyk1*/pXMJ19-*pyk2* strain under oxygen deprivation. The production of lactic acid was enhanced by increasing the concentration of Mn^2+^ and reached the highest level of 160.97 ± 1.11 mM with a yield of 82.95 ± 0.91% at 1 g/L MnSO_4_ (Fig. 5a). The production of lactic acid was improved and then gradually decreased with increasing concentrations of KCl (Fig. 5a). The highest concentration of 158.38 ± 3.57 mM with a yield of 81.76 ± 1.31% was obtained in the presence of 3 g/L KCl. These results demonstrated that the activation of Pyk2 activity by Mn^2+^ and K^+^ contributed to the increased production of lactic acid.

Fed-batch fermentation was performed in a 7.5 L fermenter to estimate the production of lactic acid at the optimal cation concentrations. The WTΔ*pyk1* strain produced 454.04 ± 5.55 mM of lactic acid with the productivity of 18.86 ± 0.21 mM/h (Fig. 5b and Table 2). To determine the distribution of carbon flux towards organic acids, the yields were expressed as a percentage of the theoretical yield (100% represented 2 mol of lactic acid, succinic acid or acetic acid per 1 mol of glucose consumed) [22, 23]. In the WTΔ*pyk1* strain, 65.60 ± 1.76% of the carbon flux was directed to lactic acid, and 25.10 ± 0.40% was directed to succinic acid. When the *pyk2* gene was overexpressed, the specific glucose uptake rate of the WTΔ*pyk1*/pXMJ19-*pyk2* strain increased by 25%, and the lactic acid production reached 669.03 ± 15.59 mM (60.27 ± 1.40 g/L) (Fig. 5c and Table 2). The lactic acid production rate of the WTΔ*pyk1*/pXMJ19-*pyk2* strain increased by 48% compared to the WTΔ*pyk1* strain. Furthermore, 77.82 ± 2.24% of the carbon flux was directed to lactic acid, and 10.57 ± 0.24% was directed to succinic acid. Taken together, these results demonstrated that the overexpression of either *pyk1* or *pyk2* increased the carbon flux into lactic acid formation under oxygen deprivation.

**Table 2:** The production of organic acids by *C. glutamicum* strains in fed-batch fermentation^a^

Strains	Glucose (mM/h)	Lactic acid (mM)	Lactic acid productivity (mM/h)	Yield^b^ (%)			
				Lactic acid	Succinic acid	Acetic acid	Total
WTΔ*pyk1*	14.38 ± 0.27	454.04 ± 5.55	18.86 ± 0.21	65.60 ± 1.76	25.10 ± 0.40	3.20 ± 0.18	93.90 ± 2.34
WTΔ*pyk1*/pXMJ19-*pyk2*	17.92 ± 0.50	669.03 ± 15.59	27.88 ± 0.65	77.82 ± 2.24	10.57 ± 0.24	7.70 ± 0.68	96.10 ± 3.16

^a^The cells were suspended to a final dry cell weight of 20 g/L in 3 L of CGXII medium with 100 mM bicarbonate. The data were derived from experiments performed at least in three times, and the *error bars* represent the standard deviations

^b^The yields were calculated as a percentage of the theoretical yield (100% represented 2 mol of lactic acid, succinic acid or acetic acid per 1 mol of glucose consumed)

## Discussion

Pyk is an important control node in response to changes in glycolysis and energy charges. Two isoenzymes of Pyk have been found to be subjected to allosteric regulation by different effectors in *E. coli* and *S. cerevisiae* [1, 8]. *C. glutamicum* was assumed to possess only one Pyk with higher activity compared to other bacterial Pyks [14, 16]. In this study, Pyk2 was identified as a functional isoenzyme in *C. glutamicum*. Because Pyk2 was Mn^2+^ and K^+^ co-dependent, marginal Pyk2 activity was detected in the *pyk1*-deficient mutant in the absence of the K^+^ cation. This indicated that the binding of K^+^ might induce the conversion of Pyk2 from the inactive conformation into the active conformation [24]. Our results confirmed that Pyk2 was activated by FBP despite the deficiency in the C-terminal domain containing the binding sites for the allosteric effector FBP in other Pyks [2, 25]. Consequently, we inferred that *C. glutamicum* Pyk2 might utilize a distinct allosteric mechanism that differed from other known FBP-activated Pyks. In *C. glutamicum*, the different allosteric activators of Pyk1 and Pyk2 made them subject to distinct metabolic regulation mechanisms similar to the regulation of the two isoenzymes in *E. coli* and *S. cerevisiae* [1, 8]. Under oxygen deprivation, the restriction of glyceraldehyde-3-phosphate dehydrogenase (GAPDH), a glycolysis rate-limiting enzyme, might result in the accumulation of FBP [12], which activated Pyk2 to direct the metabolic flux of pyruvate towards the formation of lactic acid. The allosteric effect of Pyk2 resulted in a regulatory role in pyruvate metabolism under oxygen deprivation.

The *pyk2* and *ldhA* in *C. glutamicum* were co-transcribed under aerobic conditions; therefore, *pyk2* followed the complicated regulatory mode of *ldhA*, which was subjected to dual repression under aerobic conditions mediated by the global regulator SugR and the special regulator LldR [17, 18]. SugR-mediated primary repression of *ldhA* and *pyk2* transcription was relieved in the presence of sugar to induce lactic acid formation. In turn, the generated lactic acid alleviated the LldR-mediated repression to enhance the transcription of *ldhA* and *pyk2* [26]*.* However, the mRNA level of *pyk2* was significantly higher than the mRNA level of *ldhA* under oxygen deprivation, which indicated that *pyk2* had a different transcription mode.

Under oxygen deprivation, *C. glutamicum* produces organic acids such as lactic acid, succinic acid and acetic acid from glucose with cell growth arrested [27]. The deletion or overexpression of two *pyk* genes had a significant influence on the production of organic acids. The deficiency of *pyk2* led to a decrease in the production of lactic acid and an increase in the synthesis of succinic acid. Moreover, the overexpression of *pyk2* promoted the formation of pyruvate and the production of lactic acid, whereas the metabolic flux from PEP towards oxaloacetate might decrease and led to decreased synthesis of succinic acid. Under oxygen deprivation, when the availability of pyruvate was decreased, the redundant PEP was redirected to synthesize succinic acid by PEP carboxylase, which was the predominant anaplerotic enzyme [10, 28, 29]. Our findings confirmed that *C. glutamicum* regulated the synthesis of lactic acid and succinic acid by Pyk and PEP carboxylase to maintain the intracellular NADH/NAD^+^ equilibrium under oxygen deprivation [27]. Therefore, our results suggested that Pyk is a key factor that influences the formation of lactic acid in *C. glutamicum* under oxygen deprivation.

Furthermore, Pyk2 homologs had been annotated in the genomes of *Corynebacterium*, *Mycobacterium*, *Geobacillus*, etc. The *ldhA-pyk2* cluster has been found in many representative *Corynebacterium* species (Additional file 1: Figure S3). A transcriptional unit composed of several genes encoding enzymes involved in the catalysis of related metabolic reactions has been proposed to be an effective means to rapidly adjust bacterial metabolism under different conditions [30, 31]. These results support the hypothesis that lactate dehydrogenase and Pyk2 might be interdependent and play an important role in diverting the metabolic flux into lactic acid formation in *C. glutamicum* under low oxygen tensions. Therefore, the evolution of the *ldhA-pyk2* cluster for lactic acid synthesis grants *C. glutamicum* more metabolic flexibility in response to environmental changes.

## Conclusions

In the present study, we identified that Pyk2 is a novel pyruvate kinase in *C. glutamicum* and has significantly different biochemical properties than Pyk1. The catalytic activity of Pyk2 was allosterically regulated by FBP activation. The allosteric behavior of Pyk made it an important switch point for the regulation of the glycolytic carbon flux distribution. *Pyk2* and *ldhA* were co*-*transcribed as a bicistronic mRNA under aerobic conditions. However, the mRNA level of *pyk2* in the WT strain was increased under oxygen deprivation and *pyk1* or *pyk2* deficiency decreased the production of lactic acid. The overexpression of either *pyk1* or *pyk2* increased the activity of Pyk and the production of lactic acid. The fed-batch fermentation of the *pyk2*-overexpressing strain resulted in increased lactic acid production than the parent strain, which indicated that overexpression of Pyk redirected more carbon flux into lactic acid production under oxygen deprivation.

## Methods

### Bacterial strains, plasmids, and media

The strains and plasmids used in this study are listed in Table S3 (Additional file 1: Table S3). *E. coli* EC135 [32] and BL21 (DE3) (Novagen, Germany) were used for the gene cloning and the recombinant enzyme expression experiments, respectively. The *E. coli* strains were aerobically grown at 37°C in Luria-Bertani medium [33]. The *C. glutamicum* wild-type strain ATCC 13032 (WT) was routinely cultivated in brain heart infusion (BHI) medium (37 g/L brain heart infusion with 91 g/L sorbitol) at 30°C for the genetic disruption and complementation experiments [9]. The antibiotic concentrations were 50 μg/mL kanamycin and 20 μg/mL chloramphenicol for *E. coli*, and 25 μg/mL kanamycin and 10 μg/mL chloramphenicol for *C. glutamicum*.

### Culture conditions

For the aerobic growth experiments, pre-cultures inoculated from agar plates were aerobically grown in 50 mL of CGIII medium with 4% glucose in 500 mL shake flasks on a shaker at 220 rpm for 12 h [34]. After washing the cells with CGXII minimal medium without carbon and nitrogen sources, the *C. glutamicum* strains were inoculated in 50 mL of CGXII minimal medium in 500 mL shake flasks with an initial optical density of 600 nm of approximately 1.5 (dry cell weight of 0.45 g/L) and incubated at 220 rpm [35]. A total of 100 mM glucose or 100 mM d-ribose was added as the carbon sources. The growth of the strains was assessed by measuring the OD_600_. When necessary, 0.5 mM isopropylthio-β-d-galactopyranoside (IPTG) was supplemented after inoculation.

For the oxygen-deprived growth experiments, the *C. glutamicum* strains were pre-cultured as described above for the aerobic growth experiments [34]. The cell suspension was harvested by centrifugation at 8000 × *g* and 4°C and washed with a CGXII minimal medium without carbon and nitrogen sources. The washed cells were suspended to a final dry cell weight of 10 g/L in 100 mL of CGXII minimal medium with 100 mM bicarbonate in 150 mL lidded bottles and incubated by preventing aeration and gentle agitation [10]. Oxygen deprivation was obtained using N_2_ flux. The pH was maintained at pH 7.0 by adding ammonia. Under oxygen deprivation, the growth of *C. glutamicum* was arrested. The effects of Mn^2+^ or K^+^ on fermentation were studied using MnSO_4_ (0–2 g/L) or KCl (0–15 g/L) supplementation. For the fed-batch fermentation experiments, pre-cultures inoculated from agar plates were aerobically grown in 1 L shake flasks on a shaker at 220 rpm for 12 h. Afterward, the cells were harvested by centrifugation at 8000 × *g* and 4°C, and washed with CGXII minimal medium without carbon and nitrogen sources. The washed cells were resuspended at a final dry cell weight of 20 g/L in 3 L of CGXII minimal medium with 100 mM bicarbonate and 222 mM glucose in a 7.5 L fermenter (New Brunswick BioFlo 115, Germany). N_2_ flux was flushed to maintain oxygen deprivation. When needed, 50% glucose was continuously fed to maintain the residue glucose between 0 and 50 mM. The strains were incubated at 30°C and 200 rpm, and the pH was maintained at 7.0 using a pH controller with automatic addition of an ammonia solution.

### Construction of plasmids and strains

Total genomic DNA of the WT strain was extracted according to previously described methods [36]. The oligonucleotides used in this study are shown in Table S4 (Additional file 1: Table S4). For the gene disruption experiments, the upstream and downstream homologous fragments were amplified using PCR with the primers WZ539/WZ540 and WZ541/WZ542 for the *pyk1* gene, and WZ545/WZ546 and WZ547/WZ548 for the *pyk2* gene. The amplified DNA fragments were spliced using overlap extension PCR and ligated into the suicide vector pK18*mobsacB* [37]. The plasmids were verified by DNA sequencing and then transformed into the WT strain through electroporation to generate the WTΔ*pyk1* strain, which was further verified by sequencing. Similarly, the *pyk2* gene was disrupted in the WT and WTΔ*pyk1* strains. The *C. glutamicum* and *E. coli* shuttle vector pXMJ19 was used for the gene overexpression studies. The replication origin of this vector was derived from that of pBL1 with a cryptic low copy number [38]. For the gene complementation experiments, the *C. glutamicum* strain was transformed by the plasmid pXMJ19, which contained the intact *pyk1* and *pyk2* genes. To induce the expression of the *pyk1* and *pyk2* genes, 0.5 mM IPTG was supplemented.

### Expression and purification of Pyk2

The *pyk2* gene was cloned into the vector pET-28a and expressed in the BL21 (DE3) strain. When the OD_600_ reached 0.6, the cells were induced with 0.1 mM IPTG at 16°C and harvested by centrifugation after the OD_600_ reaching 4.0. The cells were resuspended in buffer (20 mM Tris-HCl (pH 7.5), 150 mM NaCl, and 10% glycerol) and then disrupted by sonication. The supernatants were collected by centrifugation at 12,000 × *g* for 30 min at 4°C. The recombinant Pyk2 was purified using Ni^2+^-affinity chromatography (GE Healthcare, USA). The eluted protein solutions were concentrated by ultrafiltration (Merck Millipore, Germany). Subsequently, the protein solution was applied to a Resource Q column (GE Healthcare, USA), which was equilibrated with a standard buffer (20 mM Tris-HCl (pH 7.5), 1 M NaCl, and 10% glycerol). The fraction was pooled and concentrated for further analysis. The eluted protein solution was incubated with thrombin (Merck Millipore, Germany) in 1× PBS (pH 7.3) at 22 °C for 16 h, which efficiently cleaved the N-terminal His-tag. After the cleavage reaction, the reaction mixture was eluted through a Ni^2+^-affinity column to remove the His-tagged thrombin. The molecular mass of Pyk2 was analyzed using gel filtration chromatography on a Superdex 200 10/300 GL column (GE Healthcare, USA), which was equilibrated with 50 mM potassium phosphate buffer (pH 7.0) containing 150 mM NaCl. A standard curve was determined based on the elution volumes of the protein standards (GE Healthcare, USA). The purity of the purified Pyk2 was determined by SDS-PAGE. The protein concentrations were determined by the Bradford method using the Bio-Rad Protein Assay Reagent (Bio-Rad, USA).

### Enzymatic activity and property determination

The assay for Pyk activity was based on changes in the absorbance of NADH at 340 nm coupled with the l-lactate dehydrogenase [39]. The reaction mixture contained 100 mM Tris-HCl (pH 7.4), 5 mM MnSO_4_ · H_2_O, 5 mM PEP, 2 mM ADP, 100 mM KCl, 0.2 mM NADH, 6 units l-lactate dehydrogenase, and the enzyme in a total volume of 0.5 mL. To avoid a decrease in l-lactate dehydrogenase activity at extreme conditions (pH and temperature), the amount of l-lactate dehydrogenase was increased to 15 units. The reaction was initiated by the addition of PEP, and the decrease in the absorbance at 340 nm was monitored by a UV-visible spectrophotometer (Unico, USA). One unit of activity was defined as the amount of enzyme that converted 1 μmol of PEP to pyruvate per min at 25°C. To determine the effect of the pH on the enzyme activity, various buffer systems (pH 5.0–9.5) were used at 100 mM. The optimum temperature of Pyk2 was determined at temperatures ranging from 20 to 70°C. To exclude PEP hydrolysis with increasing temperatures, the relative Pyk2 activity was calculated by subtracting the background. The effects of different cations on Pyk activity were assayed by replacing Mn^2+^ with 5 mM or 10 mM of other cations (Mg^2+^, Cu^2+^, Co^2+^, Ca^2+^, Ni^2+^, Zn^2+^, Ba^2+^, Fe^2+^, Fe^3+^ or Al^3+^) or replacing K^+^ with 50 mM or 100 mM of monovalent cations (NH_4_^+^, Li^+^, or Na^+^). The enzyme activity was also measured without any cations. The data were obtained from three independent experiments.

### Analysis of kinetic properties

The enzymatic activities were assayed using various concentrations of PEP and ADP under conditions identical to those described above. To determine the kinetics, the PEP concentration was varied with the concentration of ADP at 2 mM, or the ADP concentration was varied with the concentration of PEP at 5 mM. The effects of the various effectors tested on PEP were assessed with 2 mM ADP. The formation of the product was linear throughout the period. The data were fitted to an allosteric sigmoidal kinetic model using Prism software (GraphPad, USA). The *S*_0.5_ (the substrate concentration giving one-half of *V*_max_) and *n*_H_ (Hill coefficient) values were calculated. The *k*_*cat*_ value was calculated according to the *V*_max_ and the subunit molecular weight of 67,572 Da. All data were obtained from at least three independent experiments.

### Analytical methods

Glucose was measured with an enzyme electrode glucose sensor (SBA-40D, Shandong Province Academy of Sciences, China). The dry cell weights of the *C. glutamicum* strains were calculated as one optical density unit at 600 nm to 0.3 g dry cell weight/L [40]. The specific growth rate μ (1/h) was estimated using a linear regression. The biomass yield (Y_X/S_) was deduced from the individual biomass and glucose balancing, and the specific glucose uptake rate (q_S_) was calculated from the specific growth rate and the biomass yield [41]. Organic acids were determined by high performance liquid chromatography equipped with an SB-Aq column (4.6 × 250 mm; Agilent Technologies, USA) at 210 nm. Mobile phase A (20 mM KH_2_PO_4_, pH 2.3) and mobile phase B (acetonitrile) were at a ratio of 95:5 [42]. To measure the enzyme activity, the cells were resuspended in the indicated buffer (100 mM Tris-HCl pH 7.5 and 10% glycerol) and disrupted by ultrasonic treatment at 4°C. The enzyme activity was monitored by measuring the changes in the NADH concentration at 340 nm. For Pyk, the reaction mixture was used as described above with 5 mM FBP. The Student’s *t*-test was performed with SPSS software to determine whether the difference was statistically significant.

### RNA preparation and RT-PCR analysis

Total RNA was isolated from the cells with the RNAprep Pure Cell/Bacteria Kit (Tiangen, China). The cDNA from approximately 300 ng of RNA was prepared using the specific primers listed in Table S4 (Additional file 1: Table S4) and the FastQuant RT Kit (Tiangen, China). The *rpoB* gene, which encodes RNA polymerase β subunit, was used as the reference gene [43]. The primer pair WZ1171 and WZ1156 was used to amplify the *ldhA-pyk2* region in the RT-PCR. Genomic DNA was used as a positive control in the PCR reactions. RNA reverse transcribed without reverse transcriptase was used as a negative control to exclude DNA contaminants in the RNA preparation.

The qRT-PCR (quantitative reverse transcription-PCR) was performed with the GoTaq qPCR master mix (Promega, USA) in a 20 μL mixture using the LightCycler^®^ 96 Real-Time PCR System (Roche, Switzerland). The data analysis was performed using the LightCycler^®^ 96 software (Roche, Switzerland).

### Sequence alignment

Database searches and sequence comparisons were performed with the BLAST programs at the BLAST tool on the National Center for Biotechnology Information website (https://blast.ncbi.nlm.nih.gov/Blast.cgi). Multiple sequence alignments were generated with the CLUSTAL X program [44]. Alignment of the secondary structures was produced using the high resolution X-ray crystallographic structure of *E. coli* PykF (PDB code 1PKY) as the template and ESPript 3.0 (http://espript.ibcp.fr/ESPript/ESPript/) with default settings.

## Additional file

Additional file 1: Table S1. Growth kinetics of the *pyk* mutants on glucose under aerobic conditions. **Table S2.** Kinetic parameters of the Pyk2 for substrates. **Table S3.** Strains and plasmids used in this study. **Table S4.** Primers used in this study. **Figure S1.** The map positions of the *pyk* genes and the multiple alignment of the Pyk sequences. **Figure S2.** Kinetic parameters of Pyk2 with respect to PEP at a concentration of 5 mM G6P (**a**) or AMP (**b**). **Figure S3.** Conservation of *pyk2* gene and adjacent genes in species of the *Corynebacterium* and other species. (DOCX 651 kb)

## Abbreviations

BHI: Brain heart infusion
CIT: Citrate
FBP: Fructose 1,6-bisphosphate
G6P: Glucose-6-phosphate
GAPDH: Glyceraldehyde-3-phosphate dehydrogenase
IPTG: Isopropylthio-β-d-galactopyranoside
*ldhA*: l-lactate dehydrogenase encoding gene
PEP: Phosphoenolpyruvate
PTS: Phosphotransferase transport system
Pyk: Pyruvate kinase
qRT-PCR: quantitative reverse transcription-PCR
*rpoB*: RNA polymerase β subunit encoding gene
SugR: DeoR-type transcriptional regulator
WT: Wild-type
