# Supplementary material for: A novel pyruvate kinase and its application in lactic acid production under oxygen deprivation in Corynebacterium glutamicum
Source: BMC Biotechnol. 2016 Nov 16;16:79. doi: 10.1186/s12896-016-0313-6 (PMC5112673; doi:10.1186/s12896-016-0313-6)
Supplement: Additional file 1: Table S1. — Growth kinetics of the pyk mutants on glucose under aerobic conditions. Table S2. Kinetic parameters of the Pyk2 for substrates. Table S3. Strains and plasmids used in this study. Table S4. Primers used in this study. Figure S1. The map positions of the pyk genes and the multiple alignment of the Pyk sequences. Figure S2. Kinetic parameters of Pyk2 with respect to PEP at a concentration of 5 mM G6P (a) or AMP (b). Figure S3. Conservation of pyk2 gene and adjacent genes in species of the Corynebacterium and other species. (DOCX 651 kb) [file 12896_2016_313_MOESM1_ESM.docx]

**Table S1** Growth kinetics of the *pyk* mutants on glucose under aerobic conditions

| Strains | μ (1/h) | Y_X/S_ (g/g) | q_s_ (mmol/g/h)*^a^* |
| --- | --- | --- | --- |
| WT | 0.42±0.01 | 0.49±0.02 | 4.76±0.03 |
| WTΔ*pyk1* | 0.37±0.01 | 0.49±0.04 | 4.17±0.32 |
| WTΔ*pyk2* | 0.40±0.02 | 0.50±0.03 | 4.49±0.24 |
| WTΔ*pyk1*Δ*pyk2* | 0.34±0.02 | 0.48±0.06 | 4.04±0.17 |

*^a^*The glucose consumption rate (q_s_) was calculated from the biomass yield (Y_X/S_) and the specific growth rate (μ). The data represented the averages of three replicates and ± denoted the standard deviations.

**Table S2** Kinetic parameters of the Pyk2 for substrates

| Substrates | Addition*^a^* | *S*_0.5_ (mM) | *k_cat_* (s^-1^) |
| --- | --- | --- | --- |
| ADP | - | 0.33±0.03 | 4.36±0.22 |
| PEP | - | 4.58±0.74 | 5.58±0.53 |
| PEP | FBP | 2.17±0.12 | 7.67±0.26 |
| PEP | G6P | 4.66±0.95 | 5.26±0.66 |
| PEP | AMP | 4.56±0.80 | 5.20±0.57 |
| PEP | ATP | 2.23±0.17 | 2.09±0.10 |
| PEP | CIT | 4.12±0.19 | 4.88±0.28 |

*^a^* -, no addition. The data were derived from experiments performed at least in three times, and results were means±standard errors.

**Table S3** Strains and plasmids used in this study

| Strains or plasmids | Relevant characteristics*^a^* | Source or reference |
| --- | --- | --- |
| Strains |  |  |
| *E. coli* |  |  |
| EC135 | *E. coli* TOP10∆*dcm*::*FRT* *recA*+ ∆*dam*::*FRT*, genotype of R-M systems: *mcrA*∆(*mrr-hsdRMS-mcrBC*) ∆*dcm*::*FRT* ∆*dam*::*FRT* | [[32](#_ENREF_30)] |
| BL21(DE3) | F^-^ *ompT* *gal dcm lon* *hsd*S_B_(r_B_^-^ m_B_^-^) λ(DE3) | Novagen |
| *C. glutamicum* |  |  |
| WT | ATCC 13032, the wild-type strain | ATCC |
| WT∆*pyk1* | WT with deletion in *pyk1* | This study |
| WT∆*pyk2* | WT with deletion in *pyk2* | This study |
| WT∆*pyk1*∆*pyk2* | WT with deletion in *pyk1*and *pyk2* | This study |
| WT∆*pyk1*/pXMJ19 | WT∆*pyk1* with pXMJ19 | This study |
| WT∆*pyk1*/pXMJ19-*pyk1* | WT∆*pyk1* with pXMJ19-*pyk1* | This study |
| WT∆*pyk1*/pXMJ19-*pyk2* | WT∆*pyk1* with pXMJ19-*pyk2* | This study |
| WT∆*pyk1*∆*pyk2*/pXMJ19 | WT∆*pyk1*∆*pyk2* with pXMJ19 | This study |
| WT∆*pyk1*∆*pyk2*/pXMJ19-*pyk1* | WT∆*pyk1*∆*pyk2* with pXMJ19-*pyk1* | This study |
| WT∆*pyk1*∆*pyk2*/pXMJ19-*pyk2* | WT∆*pyk1*∆*pyk2* with pXMJ19-*pyk2* | This study |
| WT/pXMJ19 | WT with pXMJ19 | This study |
| WT/pXMJ19-*pyk1* | WT with pXMJ19-*pyk1* | This study |
| WT/pXMJ19-*pyk2* | WT with pXMJ19-*pyk2* | This study |
| WT∆*pyk1*/pXMJ19-*pyk2* | WT∆*pyk1* with pXMJ19-*pyk2* | This study |
|  |  |  |
| Plasmids |  |  |
| pET-28a | Kan^r^; expression vector with an N-terminal hexahistidine affinity tag | Novagen |
| pET-28a-*pyk2* | Kan^r^; pET-28a derivative for expression of *pyk2* | This study |
| pK18*mobsacB* | Kan^r^; shuttle vector for the construction of deletion mutants | [[37](#_ENREF_34)] |
| pK18*mobsacB*-∆*pyk1* | Kan^r^; pK18*mobsacB* with DNA fragment for the deletion of *pyk1* | This study |
| pK18*mobsacB*-∆*pyk2* | Kan^r^; pK18*mobsacB* with DNA fragment for the deletion of *pyk2* | This study |
| pXMJ19 | Cm^r^; Shuttle vector for expression of proteins (*P_tac_*, *lacI*^q^) | [[38](#_ENREF_35)] |
| pXMJ19-*pyk1* | Cm^r^; pXMJ19 derivative for expression of *pyk1* | This study |
| pXMJ19-*pyk2* | Cm^r^; pXMJ19 derivative for expression of *pyk2* | This study |

*^a^*Abbreviations: Kan, kanamycin; Cm, chloramphenicol.

**Table S4** Primers used in this study

| Primer | Sequence*^a^* (5'-3') | Note |
| --- | --- | --- |
| WZ1145 | CGCCATATGAATGAGTTTGACCAGGAC *Nde*I | pET-28a-*pyk2* |
| WZ352 | CGGAATTCTTACTCTTCCCAGCTCTTCACCTTG *Eco*RI |  |
| WZ539 | CCGGAATTCGAGACTAGGTGATCTGAAGAACCCAC *Eco*RI | Up fragment for *pyk1* deletion |
| WZ540 | TGACGTACTAGGCTTTCGCTTAAATCTTTCAAAAAATGCG |  |
| WZ541 | GAAAGATTTAAGCGAAAGCCTAGTACGTCATTCCCCCTC | Down fragment for *pyk1* deletion |
| WZ542 | CCCAAGCTTCTGGGCAACTGGTTTAACCAGGAGC *Hin*dIII |  |
| WZ543 | GTTATCCTGGCGCAGGGAATTGGT | Sequencing for *pyk1* deletion |
| WZ544 | TTTCCCTCAGGATCGGTGAAGT |  |
| WZ545 | CCGGAATTCTTAGAAGCAATTCTGGAGCCTCATG *Eco*RI | Up fragment for *pyk2* deletion |
| WZ546 | GCTTAAGGAGCTCAACTCACAAAGGCGATTGGCGTTAACT |  |
| WZ547 | ATCGCCTTTGTGAGTTGAGCTCCTTAAGCTACAAACACTC | Down fragment for *pyk1* deletion |
| WZ548 | CCCAAGCTTCATGCTGGGCGAACTCTACGAAGT *Hin*dIII |  |
| WZ549 | TGCGATGAGCATAGGTATGGATAGG | Sequencing for *pyk2* deletion |
| WZ550 | CTGTCCTGGACTCCGCTCGATT |  |
| WZ1144 | TGCACTGCAGAAAGGAGGACAACCATGGGCGTGGATAGACGA *Pst*I | pXMJ19-*pyk1* |
| WZ349 | CGAGCTCTTAGAGCTTTGCAATCCTTGTGTCG *Sac*I |  |
| WZ351 | ACGCGTCGACCTTAAGGAGCTCAAATGAATGAGT *Sal*I | pXMJ19-*pyk2* |
| WZ352 | CGGAATTCTTACTCTTCCCAGCTCTTCACCTTG *Eco*RI |  |
| WZ867 | CGTACTGCTGAAGGCTCTT | *rpoB* for qRT-PCR |
| WZ868 | TTTGCTACACCATCGGACT |  |
| WZ1153 | GCGTTTGGTAGAAGACGGCAT | *pyk1* for qRT-PCR |
| WZ1154 | GCCAGTCTTCTCCGCCGCC |  |
| WZ1167 | GCCCACTTTATCCACCCTCAG | *pyk2* for qRT-PCR |
| WZ1168 | CGGCTTCGGTGGGCAGGGT |  |
| WZ1163 | TTGGCGCAGGAGATGTTGGAGTT | *ldhA* for qRT-PCR |
| WZ1164 | CCATGACGTTGCCTTCGAGTTTC |  |
| WZ1181 | GCCTTTCGGAGCCACGCTTCG | *rpoB* for RT-PCR |
| WZ868 | TTTGCTACACCATCGGACT |  |
| WZ1171 | GCATTCCGCAAATACCCTGC | *ldhA-pyk2* for RT-PCR |
| WZ1156 | ACATCTGAGGGTGGATAAAGTGGG |  |

*^a^*The restriction sites were underlined.





**Fig. S1** The map positions of the *pyk* genes and the multiple alignment of the Pyk sequences. **a.** Mapped positions of the *pyk* genes in the physical map of the genome of *C. glutamicum* ATCC 13032 (3309401 bp). The NCBI reference sequence number is NC_003450. **b.** Multiple alignment of the amino acid sequences of Pyks. The residue involved in the binding site of the monovalent cation was marked in blue ▲. **c.** Multiple alignment of the amino acid sequences of Pyks. The sequence required for PEP binding was marked in blue ●, and the conserved sequence was marked in red ▲. The sequences used in this study were Ec_PykF (*E. coli*, WP_001295403), Ec_PykA (*E. coli*, WP_000091148), Cg_Pyk1 (*C. glutamicum*, WP_011014873) and Cg_Pyk2 (*C. glutamicum*, WP_011015482).


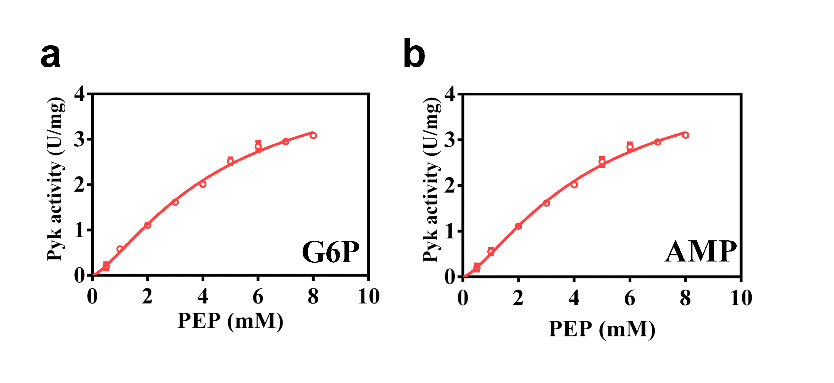


**Fig. S2** Kinetic parameters of Pyk2 with respect to PEP at a concentration of 5 mM G6P (**a**) or AMP (**b**). The data points were fitted using Prism (GraphPad). The experiments were performed at least in three times, and the *error bars* represent the standard error of the regression.





**Fig. S3** Conservation of *pyk2* and adjacent genes in the genome of *Corynebacterium* and other species. *pyk* (pyruvate kinase), *ldh* (lactate dehydrogenase), *gntP* (gluconate permease), *sod* (superoxide dismutase), *glpQ* (glycerophosphoryl diester phosphodiesterase). Arrows indicated the direction of gene transcription.
